# Description of Child and Adolescent Beverage and Anthropometric Measures According to Adolescent Beverage Patterns

**DOI:** 10.3390/nu10080958

**Published:** 2018-07-25

**Authors:** Teresa A. Marshall, Alexandra M. Curtis, Joseph E. Cavanaugh, John M. VanBuren, John J. Warren, Steven M. Levy

**Affiliations:** 1 Department of Preventive & Community Dentistry, College of Dentistry, The University of Iowa, Iowa City, IA 52242, USA; john-warren@uiowa.edu; 2 Department of Biostatistics, College of Public Health, The University of Iowa, Iowa City, IA 52242, USA; alexandra-curtis@uiowa.edu; 3 Department of Statistics and Actuarial Science, College of Liberal Arts and Sciences, The University of Iowa, Iowa City, IA 52242, USA; joe-cavanaugh@uiowa.edu; 4 Department of Pediatrics, Division of Critical Care, School of Medicine, The University of Utah, Salt Lake City, UT 84132, USA; john.vanburen@hsc.utah.edu; 5 Department of Preventive & Community Dentistry, College of Dentistry, and Department of Epidemiology, College of Public Health, The University of Iowa, Iowa City, IA 52242, USA; steven-levy@uiowa.edu

**Keywords:** beverage, height, body mass index, children

## Abstract

Our objective is to retrospectively describe longitudinal beverage intakes and anthropometric measures according to adolescent beverage patterns. Data were collected from Iowa Fluoride Study participants (*n* = 369) using beverage questionnaires at ages 2–17 years. Weight and height were measured at ages 5, 9, 13 and 17 years. Cluster analyses were used to identify age 13- to 17-year beverage patterns. Treating age and beverage cluster as explanatory factors, sex-specific generalized linear mixed models were used to identify when differences in beverage intakes and anthropometric measures began. Predominant beverage intakes were higher in each of the corresponding clusters by 9–12.5 years; females with high milk intakes during adolescence and males with high 100% juice or sugar-sweetened beverage intakes during adolescence reported higher intakes of that beverage beginning at 2–4.7 years. Females and males in the 100% juice cluster had lower weights than other clusters beginning at 13 years, while females and males in the neutral cluster were shorter beginning at 13 years. Females in the water/sugar-free beverage cluster had higher body mass indices (BMIs) beginning at 9 years. Females and males in the 100% juice cluster had lower BMIs beginning at 5 and 9 years, respectively. Childhood beverage intakes and growth patterns differ according to adolescent beverage patterns.

## 1. Introduction

Optimal growth is characterized by achievement of genetic height potential, normal body mass index, normal cognitive function and emotional well-being. Achievement of optimal growth is associated with an enriching environment, including adequate and appropriate nutrition, sleep, housing, and emotional support [1,2,3], and limited exposure to environmental stressors such as lead poisoning [4]. Nutritional challenges to optimal growth are associated with recognized growth insults. For example, excessive energy intake contributes to obesity in developed countries [5,6], while inadequate energy or protein intakes contribute to stunting (i.e., a linear growth deficit) in developing countries [7]. 

Historically, pediatric nutritional science focused on the identification of nutrients needed for optimal growth and development. In recent years, attention has turned to understanding dietary contributors to obesity. The prevalence and incidence of obesity in the United States (U.S.) have increased dramatically during the past 50 years. Although rates of overweight and obesity appear to have stabilized recently among children aged 6–11 years, rates continue to increase among children aged 2–5 and 12–19 years [8,9,10]. Understanding dietary risk factors for obesity is important given the significant chronic disease associated with adolescent and adult obesity [11,12]. 

While excessive energy intake is considered to be a primary cause of obesity, recent investigations have focused on added sugars intake, particularly from sugar-sweetened beverages (SSB), although the evidence is inconclusive [5,6,13]. Investigation of individual beverages is appropriate; however, most individuals consume a variety of beverages. Unfortunately, investigation of beverage patterns and obesity has received much less attention. We recently investigated associations among adolescent beverage patterns and age 17-year anthropometric measures in a birth cohort [14]. We reported that BMIs were higher on average for members of the water/sugar-free beverage (SFB) cluster, milk cluster, neutral (no dominant beverage) cluster and SSB cluster than for members of the 100% juice cluster. 

Additionally, we reported that age 17-year mean heights differed among beverage patterns [14]. Specifically, females belonging to the neutral beverage cluster were shorter than females in the milk and SSB clusters. Males belonging to the neutral beverage cluster were shorter than males in the milk and water/SFB clusters. While mean heights of all beverage clusters were well within the normal range of the U.S. Centers for Disease Control and Prevention’s growth charts [15], the differences among beverage clusters were unexpected. 

Our search of the scientific literature for reports of associations of dietary variables, in particular beverage intakes, with adolescent heights in developed countries was fruitless. We feel that understanding the origin of the observed anthropometric differences, as well as concurrent beverage patterns, among beverage clusters is necessary for public health initiatives addressing optimal growth. Therefore, the objective of the current study is to retrospectively describe beverage intakes and anthropometric measures of the previously-identified beverage clusters. 

## 2. Materials and Methods 

### 2.1. Data Collection 

These descriptive analyses used data collected as part of the Iowa Fluoride Study (IFS), which included longitudinal investigations of fluoride, dietary exposures and oral health [14,16,17]. Demographic data were collected at recruitment and in 2007. Beverage intakes were collected by questionnaire at 3 to 6 months intervals following birth. Anthropometric measures were obtained at clinic visits when study participants were approximately ages 5, 9, 13 and 17 years. All components of the IFS were approved by the Institutional Review Board at the University of Iowa (#199112665). Written informed consent was obtained from mothers at recruitment and from parents at clinic visits, and written assent was obtained from study participants beginning at age 13 years. 

### 2.2. Participants 

Mothers were recruited at the time of their infants’ births for their children’s participation in the IFS from 1992–1995. Inclusion criteria for the current study required participation in the age 17 clinic exam (*n* = 465), return of at least one questionnaire completed between ages 13.0 years and 14.0 years and a second questionnaire between 16.0 and 17.0 years for beverage cluster determination (*n* = 369, and a recorded outcome variable for at least one of the four time points (*n* = 369), with *n* = 369 the final sample size for these analysis. 

### 2.3. Beverage Intakes 

Beverage intakes were identified using validated beverage frequency questionnaires completed at 3–6 months intervals between ages 2 and 17 years. [16,17]. The questionnaires queried whether a particular type of beverage was consumed during the previous week, and, if consumed, the frequency and quantity of consumption. Prior to age 9, beverage questions queried flavored and unflavored milk, ready-to-drink 100% juice and juice drinks, soft drinks, reconstituted powdered beverages and water. After age 9, 100% juice and juice drinks were queried separately, and sports drinks, coffee and alcohol were also queried. If a beverage was consumed, then the participant was asked to provide detailed product information, including the brand, type, and flavor. Beverages were collapsed into four categories: water and other sugar-free beverages (water/SFBs), milk, 100% juice, and sugar-sweetened beverages (SSBs). Mean daily beverage intakes were averaged from all available questionnaires at ages 2–4.7 (*n* = 369), 5–8.5 (*n* = 368), 9–12.5 (*n* = 366), and 13–17 (*n* = 369) years. Age 13 to 17 years averages of the four beverage categories were used for clustering. For the beverage intake analysis, all subjects (*n* = 369) with cluster assignment were included in the model. However, for data corresponding to a particular age range to be included in the model, a subject needed to have at least one questionnaire returned during that age range.

### 2.4. Anthropometric Measures 

Weight and height were measured in light clothing without shoes at age 5 (*n* = 351), 9 (*n* = 343), 13 (*n* = 368) and 17 (*n* = 367) clinic visits. Weight was measured using a standard physician’s scale, and height was measured using a stadiometer. Body mass index (kg/m^2^) was calculated from these measurements. 

### 2.5. Statistical Analyses 

Identification of beverage cluster membership was previously described [14]. Using the “stats” package in R (version 3.3.2, Statistical Computing, Vienna, Austria), Ward’s hierarchical method was employed to partition the subjects into clusters [18,19]. In brief, five beverage pattern clusters were created based on the individuals’ four beverage intakes from ages 13–17. The clusters were named for the predominant beverage consumed and included: 100% juice, milk, water/SFBs, SSBs and a ‘neutral’ cluster indicating relatively low intakes of all beverage types.

The outcome variables were beverage intakes and anthropometric measures throughout childhood and adolescence. For each outcome of interest, generalized linear mixed models (GLMMs) were fit to characterize the mean outcome longitudinally. The predictor variables for each model were the beverage cluster, age and the interaction between beverage cluster and age. The predictor variable for age was coded as a categorical variable with four time points at ages 5, 9, 13, and 17. For each GLMM, the conditional distribution and the link function were specified based on the empirical distribution of the outcome variable. Separate models were constructed for male and female subjects for each outcome of interest. 

The distribution of beverage intakes (i.e., juice intake) was right skewed, so a gamma distribution and a log link function were used to model these outcome variables. Since the gamma distribution cannot be used to model outcomes of zero and some subjects had beverage intakes of 0 for a beverage category, 0.001 was added to each observation for the beverage intakes so that subjects with beverage intakes of 0 would be included in the model. The distributions of weight and BMI were also right-skewed, so a gamma distribution was also used, but an identity link function was chosen due to improved penalized model fit over the log link function. Height was normally distributed, and linear mixed models with the identity link function were used. 

GLMMs with subject-specific random intercepts were fit using the GLIMMIX procedure in SAS (version 9.4; SAS Institute Inc., Cary, NC, USA). The Laplace approximation was employed to fit the models based on the marginal likelihood. Model-based mean estimates and their corresponding standard errors were obtained for each cluster at each time point. If variance estimates for the random effects were non-positive, random effects were excluded from the model.

Model-based mean daily beverage intakes and anthropometric measures and their 95% confidence intervals were calculated based on the fitted GLMMs. GLMMs have a subject-specific interpretation, so the model-based means can be interpreted as the expected value of the outcome variable for the typical female or male subject in that particular cluster (i.e., a subject having a random effect of zero). In the interpretation of results, we were primarily interested in clinically meaningful differences in the model-based mean estimates. We place emphasis on model-based mean estimates where the 95% confidence intervals do not overlap. Meaningful differences between cluster means with overlapping confidence intervals were also considered, although in such instances, the variability of the model-based estimates necessitates caution when making generalizing conclusions.

## 3. Results

### 3.1. Demographics 

The mean ± standard deviation (SD) age of participants meeting inclusion criteria was 17.7 ± 0.7 years at the age 17-year clinic exam; these values were the same for females and males. Forty-eight percent of subjects were male; 50% had mothers with a 4-year college degree or higher in 2007; and 68% were raised in households with an annual income of ≥$60,000 in 2007 (Supplemental Table S1). Most adolescents were non-Hispanic white (97.6%).

### 3.2. Beverage Intakes 

**Figure 1:**
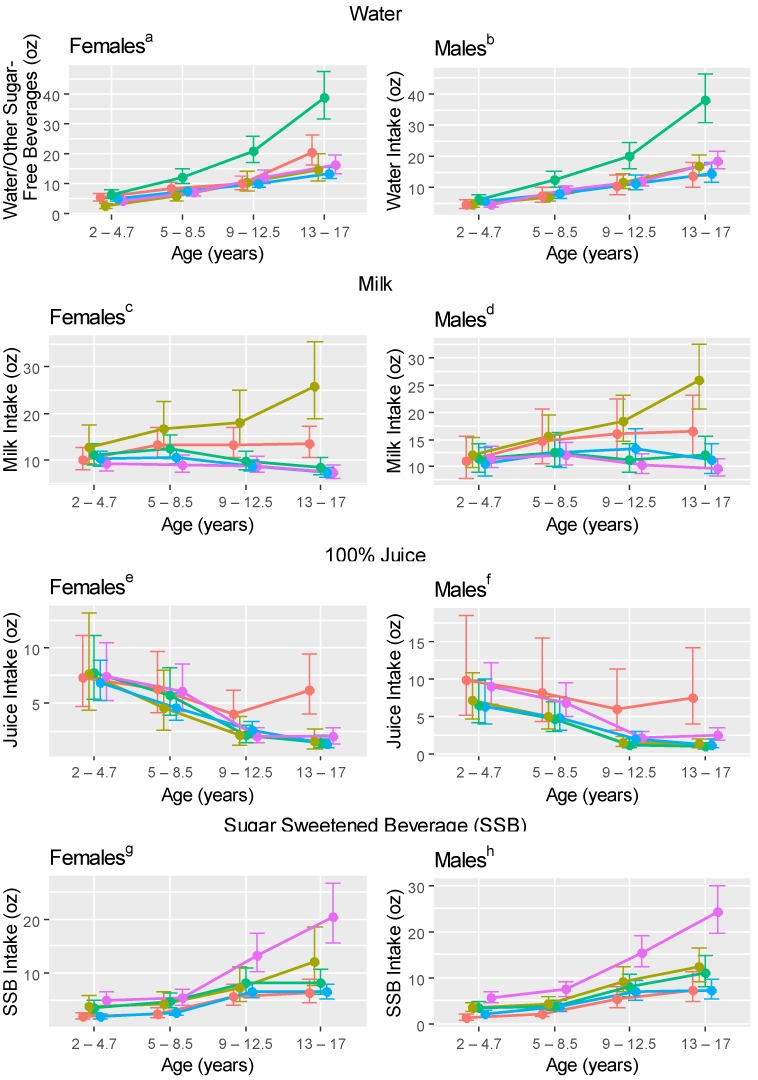
Age 2–4.7, 5–8.5, 9–12.5 and 13–17 year ^i^ mean ^j^ (95% confidence interval; CI) daily beverage intakes of age 13–to 17–year Iowa Fluoride Study beverage clusters (*n* = 369; 193 female and 176 male). Red–100% Juice Cluster, Gold–Milk Cluster, Green–Water, Blue–Neutral (no dominant beverage) Cluster, Purple–Sugar-Sweetened Beverage Cluster. ^a,b^ Model-based mean water/SFB intakes and 95% CIs for females and males in each beverage cluster. ^c,d^ Model-based mean milk intakes and 95% CIs for females and males in each beverage cluster. ^e,f^ Model-based mean 100% juice intakes and 95% CIs for females and males in each beverage cluster. ^g,h^ Model-based mean SSB intakes and 95% CIs for females and males in each beverage cluster. ^i^ All values represent the same approximate age; points are shifted for ease of reading. ^j^ Model-based mean estimates. In order to satisfy the positivity assumption of the gamma distribution, 0.001 was added to all observations to avoid outcome variable values of 0.

Differences in model-based mean beverage intakes among clusters are summarized in Table 1, and model-based mean daily beverage intakes are provided in Figure 1. and Supplemental Table S2 For example, looking at the first rows of Table 1, both females and males in the water/SFB cluster reported higher intakes of water/SFB compared to other clusters beginning at 5–8.5 years. Females in the milk cluster reported higher milk intakes compared to other clusters at all ages, while females in the juice cluster reported higher milk intakes compared to the water/SFB/SSB and neutral clusters beginning at 9–12.5 years. Males in the milk and juice clusters also reported higher milk intakes than members of the water/SFB, SSB and neutral clusters beginning at 5–8.5 years. Females and males in the juice cluster reported higher intakes of 100% juice compared to other clusters beginning at 9–12.5 and 2–4.7 years, respectively. Males in the SSB cluster also reported higher juice intakes compared to the milk, water/SFB and neutral clusters at 2–4.7, 5–8.5 and 13–17 years. Females and males in the SSB cluster reported higher intakes of SSB than members of other clusters beginning at 9–12.5 and 2–4.7 years, respectively. In summary, differences in beverage intakes among beverage clusters most commonly occurred between ages 9–12.5 and 5–8.5 years for females and males, respectively. 

### 3.3. Anthropometrics 

**Figure 2:**
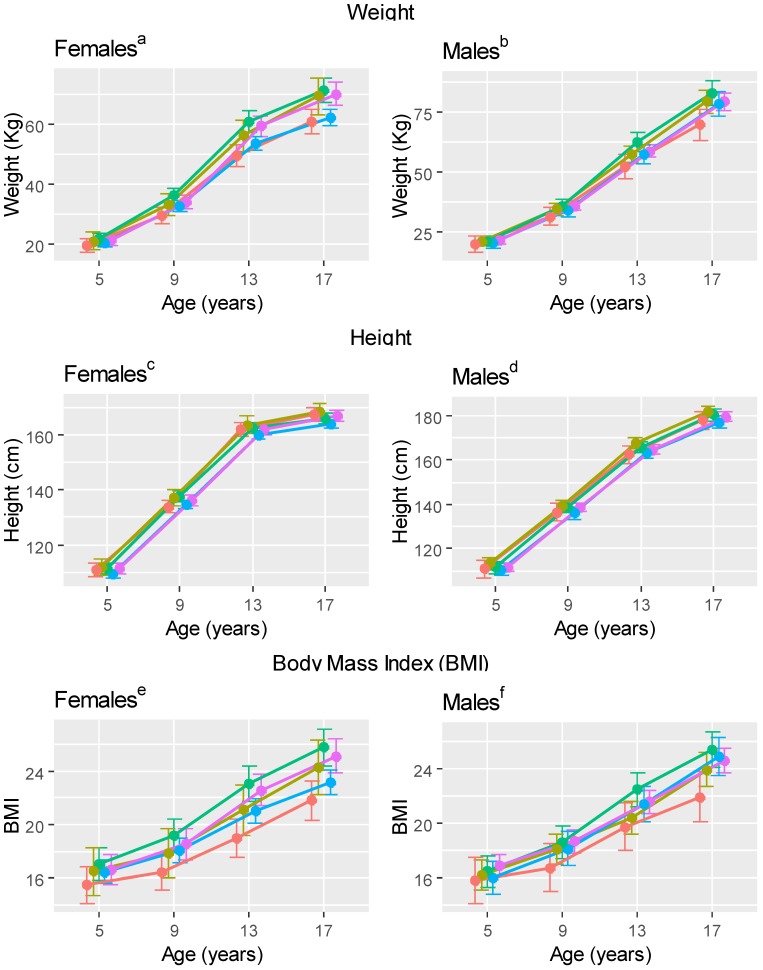
Age 5, 9, 13 and 17-year ^g^ mean ^h^ (95% CI) anthropometric measures of age 13 to 17 years Iowa Fluoride Study beverage clusters (*n* = 369; 193 female and 176 male). Red–100% Juice Cluster, Gold–Milk Cluster, Green–Water, Blue–Neutral (no dominant beverage) Cluster, Purple–Sugar-Sweetened Beverage Cluster. ^a,b^ Model-based mean weight and 95% CIs for females and males in each beverage cluster. ^c,d^ Model-based mean height and 95% CIs for females and males in each beverage cluster. ^e,f^ Model-based mean BMI and 95% CIs for females and males in each beverage cluster. ^g^ All values represent the same approximate age; points are shifted for ease of reading. ^h^ Model-based mean estimates.

Differences in model-based mean anthropometric measures by age among clusters are summarized in Table 2, with model-based mean values reported in Figure 2 and Supplemental Table S3. For example, looking at the first row of Table 2, we see females in the juice and neutral clusters weighed less (i.e., lower mean weight values) compared to the milk, water/SFB and SSB clusters beginning at the 13-year examination, while males in the juice cluster weighed less compared to all other clusters beginning at 13 years. Females in the neutral cluster were shorter compared to other clusters beginning at 13 years, while males in the neutral cluster were shorter compared to the water/SFB, milk and SSB clusters beginning at 13 years. Females in the water/SFB cluster had higher BMIs compared to other clusters beginning at age 9 years. Females and males in the juice cluster had lower BMIs compared to other clusters beginning at 5 and 9 years, respectively. Differences in weight and height measures among beverage clusters generally occurred by 13 years, while differences in BMI were noted at 9 years for both sexes. 

An additional set of models for the anthropometric analysis were fit which adjusted for mother's education level and household income in 2007 in order to address possible confounding. However, the results from these models communicate similar conclusions about the association between beverage cluster and height, weight, and BMI as the unadjusted models. The unadjusted models are presented for simplicity of interpretation. 

## 4. Discussion

Herein, we described beverage intakes and anthropometric measures according to adolescent beverage patterns. Discrimination of predominant beverage intakes was apparent by 9–12.5 years for all clusters, with beverage intake preferences for female members of the milk cluster and male members of the juice and SSB clusters noted at 2–4.7 years. Differences in weight and height appeared beginning at 13 years, while differences in BMI were noted earlier. These results challenge the general assumption that overt and/or subtle nutritional deficiencies are only of historic interest for those living in western societies [20]. The divergent growth parameters with age according to adolescent beverage patterns suggests that achievement of optimal growth is not a certainty in the U.S.

The consistent link between early and subsequent high SSB intakes by male members of the SSB cluster is consistent with previous observations of SSB intakes. Norwegian boys and girls were categorized by low, medium or high SSB intake frequencies at 18 months, and they mostly remained in their respective frequency categorizations at ages 3 and 7 years [21]. Age 8 to 14 years SSB intakes of Mexican children were positively-associated with preschool SSB intakes; tertile categorization of age 8 to 14 years intakes mimicked preschool age categories [22]. SSB introduction during infancy and mean SSB intake at 10–12 months were associated with increased likelihood of SSB intake at 6 years in a cohort of U.S. children [23]. A suggested upper limit (based on added sugars) for adult SSB intakes is 8 oz/day [24]; this recommendation was exceeded by typical female and male members of the milk, water and SSB clusters at 17 years of age. These data, combined with the existing literature, suggest that strategies to reduce adolescent SSB intakes should begin early in childhood. 

Members of all clusters reported higher mean 100% juice and/or juice drink intakes for ages 2–4.7 years than currently recommended by the American Academy of Pediatrics (i.e., ≤4 oz ages 1–3 years and 4–6 oz ages 4–6 years) [25]. Members of the juice cluster were generally within recommended intakes (i.e., ≤8 oz/day) at older ages. Juice intakes were generally consistent with intakes reported for young children participating in the National Health and Nutrition Examination Survey during the past several decades [26,27]. Sonneville et al. reported that children who drank 8 oz or more of 100% juice and SSBs at age one year had higher intakes of juice and SSB intakes at older ages [28]. Similarly, male members of our SSB cluster had higher intakes of juice at multiple ages than members of the milk, water/SFB and neutral clusters. 

Members of the milk cluster had higher milk intakes than most other clusters during adolescence. Only members of our milk cluster had a mean intake consistent with the recommended 2.5–3.0 cups of milk at age 17 years [29]. Maillot et al. reported that NHANES participants aged 4–19 years who consumed primarily milk and 100% juice had higher Healthy Eating Indices than participants consuming primarily milk (no 100% juice), primarily 100% juice (no milk) or other beverage patterns [30]. Public health initiatives that encourage adequate milk and age appropriate 100% juice intakes remain appropriate.

Differences in height were first noted at 13 years of age. The differences were modest–mean heights of members of the neutral cluster were 4–5 cm shorter than their tallest peer cluster at age 17 years. Members of the neutral clusters reported low total beverage intakes throughout adolescence, but intakes were not necessarily lower than other clusters. The clinical relevance of the height difference is unclear and may or may not be related to diet. Furthermore, while the difference in height became apparent during adolescence, we are unable to determine if factors contributing to the height difference occurred during adolescence, at earlier points in time, or both. The height differences do not meet criteria for stunting associated with cognitive deficits; however, relationships among slight linear growth retardation and cognitive, physical and emotional health are unknown. The few reports of beverage intakes and height in western cultures suggest that low milk intakes are associated with shorter stature in New Zealand children and lower height velocities in Japanese children [31,32].

Mean BMIs of female members of the milk, water and SSB clusters and male members of the water, neutral and SSB clusters met the Centers for Disease Control and Prevention’s cutoff for ‘overweight’ at age 17 years. Female members of the neutral cluster had BMIs that were lower than members of the milk, water/SFB and SSB clusters, while male members of the neutral cluster had BMIs similar to those of the milk, water/SFB and SSB clusters. These results are not consistent with previous investigations reporting associations between SSBs and obesity, or liquid energy and risk of obesity [33,34].

The importance of early childhood nutrition on future growth and development is well-accepted, and it has resulted in the establishment of public health programs (i.e., Women, Infant & Children, Supplemental Nutrition Assistance Program, National School Lunch Program,) targeting adequate and appropriate nutrition for children of low socioeconomic status. As children age, their vulnerability to nutritional insults diminishes; fewer programs target older children and adolescents. Our study participants are reasonably well off financially, with most above food insecurity thresholds, suggesting that other factors are at play. Education, knowledge, early habits, susceptibility to marketing and too much independence are factors that could impact food choices and behaviors of our adolescents. Few public health programs address diet and nutrition education of adolescents, perhaps increasing their vulnerability to dietary insults. The current study was not designed to investigate environmental contributors to food choices or diet quality; however, the results suggest that beverage patterns–perhaps a reflection of overall food choices–are associated with poor diet quality, obesity and linear growth retardation. 

Limitations must be considered when interpreting our results. Both diet and beverage intakes were caregiver-reported at younger ages and self-reported at older ages and might not reflect actual intakes. Beverage questionnaires combined 100% juice and juice drinks prior to age 9 years; thus, juice drinks are included with 100% juice for 2–4.7 and 5–8.5 years. Inclusion of juice drinks with 100% juice prior to age 9 may have limited our ability to identify healthy effects of 100% juice and/or unhealthy effects of SSBs at early ages. The sample is small in size and self-selected. The population is mostly white, reasonably well-educated and reasonably wealthy, and is not representative of other U.S. or international populations. The study also has several strengths, including a loyal, long-standing cohort. Beverage intakes were queried using validated instruments at multiple time points throughout life, which provides a better estimate of actual intake than does a single measure. Longitudinal data enable us to examine patterns of beverage intakes and anthropometric measures over time without undue concerns regarding a cohort effect. 

## 5. Conclusions

After clustering subjects according to adolescent beverage patterns, retrospective differences in beverage intakes were observed across childhood. SSB intakes have been associated with obesity; however, observed differences in BMI are not consistent with previous literature. Furthermore, observed differences in height among beverage patterns were unexpected. While beverage patterns might reflect food choice differences, early and sustained nutrition education, ready access to appropriate foods and beverages and additional investigation are necessary to improve the health and well-being of adolescents.

## Figures and Tables

**Table 1:** Summary of sex- and age-specific findings pertaining to mean beverage intake differences ^†^ among age 13 to 17 years Iowa Fluoride Study beverage clusters (*n* = 369; 193 female and 176 male).

Beverage Category	Sex	Ages(years)	Finding	
			Higher Mean Values	Lower Mean Values
Water/SFB *	Female	5–8.5	Water/SFB cluster	All other clusters
		9–12.5		
		13–17		
	Male	5–8.5	Water/SFB cluster	All other clusters
		9–12.5		
		13–17		
Milk	Female	2–4.7	Milk cluster	All other clusters
		5–8.5		
		9–12.5		
		13–17		
	Female	9–12.5	Juice cluster	Water/SFB cluster SSB ^†^ cluster Neutral cluster
		13–17		
	Male	5–8.5	Milk clusterJuice cluster	Water/SFB cluster SSB cluster Neutral cluster
		9–12.5		
		13–17		
100% Juice	Female	9–12.5	Juice cluster	All other clusters
		13–17		
	Male	2–4.7	Juice cluster	All other clusters
		5–8.5		
		9–12.5		
		13–17		
	Male	2–4.7	SSB cluster	Milk cluster Water/SFB cluster Neutral cluster
		5–8.5		
		13–17		
SSBs *	Female	9–12.5	SSB cluster	All other clusters
		13–17		
	Male	2–4.7	SSB cluster	All other clusters
		5–8.5		
		9–12.5		
		13–17		

* SFB, sugar-free beverages; SSB = sugar-sweetened beverages. ^†^ Emphasis was placed on estimates where the 95% confidence intervals do not overlap. Meaningful differences between cluster means with overlapping confidence intervals were also considered, although the variability of the estimates necessitates caution when making generalizing conclusions.

**Table 2:** Summary of sex and age-specific findings pertaining to mean anthropometric differences ^†^ among age 13 to 17 years Iowa Fluoride Study beverage clusters (*n* = 369; 193 female and 176 male).

Variable	Sex	Age (year)	Finding	
			Higher Mean Values	Lower Mean Values
Weight	Females	13	Milk cluster Water/SFB * cluster SSB * cluster	Juice cluster Neutral cluster
		17		
	Males	13	All other clusters	Juice cluster
		17		
Height	Females	13	All other clusters	Neutral cluster
		17		
	Males	13	Milk cluster Water/SFB cluster SSB cluster	Neutral cluster
		17		
Body Mass Index	Females	9	Water/SFB cluster	All other clusters
		13		
		17		
		5	All other clusters	Juice cluster
		9		
		13		
		17		
	Males	9	All other clusters	Juice cluster
		13		
		17		

* SFB, sugar-free beverages; SSB = sugar-sweetened beverages. ^†^ Emphasis was placed on estimates where the 95% confidence intervals do not overlap. Meaningful differences between cluster means with overlapping confidence intervals were also considered, although the variability of the estimates necessitates caution when making generalizing conclusions.

## Supplementary Materials

The following are available online at http://www.mdpi.com/2072-6643/10/8/958/s1. Table S1: Socioeconomic status of Iowa Fluoride subjects by age 13- to 17-year beverage clusters. Table S2: Model based mean (95% confidence interval) daily beverage intakes of Iowa Fluoride subjects by age 13- to 17-year beverage cluster (*n* = 369). Table S3: Model-based mean (95% confidence interval) anthropometric measures of Iowa Fluoride subjects by age 13- 17-year beverage cluster (*n* = 365).
