# Supplementary material for: Description of Child and Adolescent Beverage and Anthropometric Measures According to Adolescent Beverage Patterns
Source: Nutrients. 2018 Jul 25;10(8):958. doi: 10.3390/nu10080958 (PMC6115990; doi:10.3390/nu10080958)
Supplement: Supplementary file 1 [file nutrients-10-00958-s001.pdf]

Supplemental Table 1: Socioeconomic status of Iowa Fluoride subjects by age 13- to 17-year beverage clusters.

			Cluster Group									
	All Subjects		100% Juice		Milk		Water		Neutral		SSB	
Sex	M	F	M	F	M	F	M	F	M	F	M	F
Number of Subjects	176	193	15	27	35	16	33	37	30	71	63	42
	Socioeconomic variable											
Income ≥ $60,000 at Recruitment (%)*	17.0	19.2	13.3	25.9	17.1	12.5	6.1	24.3	26.7	18.3	19.0	14.3
Mother with ≥ 4 year degree at Recruitment (%)*	31.1	36.0	53.3	48.0	41.2	18.8	26.7	40.5	29.6	41.4	23.0	22.0
Income ≥ $60,000 in 2007 (%)*	70.4	66.0	86.7	76.9	73.5	80.0	62.5	64.9	72.4	66.2	67.8	53.9
Mother with ≥ 4 year degree in 2007 (%)*	47.4	52.7	80.0	77.8	51.4	53.3	43.8	48.7	55.2	54.3	35.5	35.9

*Percentages are the percent of subjects in a particular column of the table (based on dietary cluster and sex) who meet the socioeconomic status criteria.

Supplemental Table 2: Model-based mean (95% confidence interval) daily beverage intakes of Iowa Fluoride subjects by age 13- to 17-year beverage cluster (n=369).

Variable	Age (years)	Cluster Group									
		100% Juice		Milk		Water/SFB[a]		Neutral		SSB[b]	
		F	M	F	M	F	M	F	M	F	M
n		27	15	16	35	37	33	71	30	42	63
Water/SFB (oz)	2-4.7	5.4 (4.3,6.9)	4.6 (3.4,6.2)	2.7 (2.0,3.6)	4.4 (3.6,5.4)	6.6 (5.4,8.1)	6.2 (5.1,7.6)	5.0 (4.3,5.8)	5.6 (4.5,6.9)	4.2 (3.4,5.0)	4.5 (3.9,5.2)
	5-8.5	8.5 (6.7,10.7)	7.4 (5.5,10.1)	5.9 (4.4,8.1)	7.0 (5.8,8.6)	12.4 (10.1,15.1)	12.3 (10.1,15.1)	7.6 (6.6,8.8)	8.0 (6.5,9.9)	7.1 (5.8,8.6)	9.2 (7.9,10.7)
	9-12.5	10.1 (8.0,12.8)	10.4 (7.7,14.1)	10.6 (7.8,14.4)	11.7 (9.6,14.3)	21.1 (17.2,25.8)	19.9 (16.2,24.4)	10.2 (8.8,11.8)	11.3 (9.1,14.0)	12.2 (10.1,14.7)	12.1 (10.5,14.1)
	13-17	20.8 (16.4,26.4)	13.5 (10.0,18.2)	15.0 (11.0,20.4)	16.7 (13.7,20.3)	38.9 (31.8,47.6)	37.8 (30.9,46.3)	13.6 (11.8,15.8)	14.6 (11.8,18.0)	16.3 (13.5,19.7)	18.6 (16.0,21.5)
Milk (oz)	2-4.67	10.0 (7.9,12.8)	11.0 (7.8,15.5)	12.7 (9.2,17.4)	12.3 (9.8,15.3)	11.0 (8.9,13.5)	11.4 (9.0,14.3)	10.2 (8.8,11.9)	10.6 (8.3,13.5)	9.3 (7.6,11.3)	11.6 (9.8,13.7)
	5-8.5	13.3 (10.4,17.0)	14.7 (10.5,20.7)	16.6 (12.1,22.7)	15.6 (12.4,19.4)	12.5 (10.2,15.4)	12.6 (10.0,15.9)	10.5 (9.1,12.2)	12.7 (10.0,16.2)	9.0 (7.4,11.0)	12.1 (10.3,14.3)
	9-12.5	13.3 (10.5,17.0)	16.0 (11.4,22.5)	18.1 (13.2,24.9)	18.4 (14.6,23.1)	9.7 (7.9,12.0)	11.3 (9.0,14.3)	8.7 (7.5,10.1)	13.3 (10.4,16.9)	8.8 (7.2,10.7)	10.4 (8.8,12.3)
	13-17	13.5 (10.6,17.2)	16.4 (11.7,23.1)	25.8 (18.8,35.3)	25.9 (20.7,32.4)	8.5 (6.9,10.4)	12.3 (9.7,15.5)	7.3 (6.3,8.5)	11.2 (8.8,14.3)	7.3 (6.0,8.9)	9.7 (8.2,11.6)
100% Juice (oz); includes juice drinks before age 9 years	2-4.67	7.3 (4.7,11.1)	9.9 (5.3,18.6)	7.6 (4.4,13.2)	7.2 (4.8,10.9)	7.8 (5.4,11.2)	6.5 (4.3,10.0)	6.8 (5.3,8.9)	6.4 (4.1,10.0)	7.5 (5.3,10.5)	9.0 (6.7,12.3)
	5-8.5	6.3 (4.1,9.7)	8.3 (4.4,15.5)	4.6 (2.6,8.0)	5.1 (3.4,7.7)	5.7 (3.9,8.2)	4.7 (3.1,7.1)	4.6 (3.5,5.9)	5.0 (3.2,7.8)	6.1 (4.3,8.6)	7.0 (5.1,9.5)

	9-12.5	4.0 (2.6,6.1)	6.1 (3.2,11.4)	2.1 (1.2,3.8)	1.6 (1.0,2.4)	2.1 (1.5,3.0)	1.3 (0.9,2.0)	2.5 (1.9,3.4)	2.0 (1.3,3.1)	2.0 (1.4,2.8)	2.3 (1.7,3.1)
	13-17	6.2 (4.0,9.5)	7.6 (4.1,14.3)	1.5 (0.8,2.7)	1.4 (0.9,2.1)	1.4 (1.0,2.1)	1.0 (0.7,1.6)	1.3 (1.0,1.7)	1.3 (0.8,2.0)	1.9 (1.4,2.8)	2.6 (1.9,3.5)
SSBs (oz)	2-4.67	2.0 (1.4,2.8)	1.3 (0.8,2.0)	3.9 (2.5,6.0)	3.4 (2.5,4.6)	3.7 (2.7,4.9)	3.5 (2.6,4.7)	2.0 (1.6,2.4)	2.1 (1.5,2.9)	5.1 (3.9,6.7)	5.7 (4.6,7.1)
	5-8.5	2.5 (1.8,3.5)	2.2 (1.4,3.4)	4.2 (2.7,6.5)	4.3 (3.2,5.7)	4.8 (3.6,6.4)	3.7 (2.8,5.0)	2.6 (2.1,3.2)	3.7 (2.7,5.1)	5.4 (4.1,7.0)	7.4 (6.0,9.1)
	9-12.5	5.8 (4.1,8.0)	5.2 (3.4,8.0)	7.3 (4.8,11.3)	9.2 (6.8,12.4)	8.2 (6.2,10.9)	7.9 (5.9,10.6)	6.6 (5.4,8.2)	6.9 (5.0,9.4)	13.3 (10.2,17.4)	15.4 (12.4,19.0)
	13-17	6.3 (4.5,8.8)	7.3 (4.7,11.3)	12.0 (7.8,18.6)	12.2 (9.2,16.3)	8.1 (6.1,10.8)	10.9 (8.1,14.7)	6.5 (5.3,8.0)	7.1 (5.2,9.7)	20.4 (15.6,26.6)	24.2 (19.6,30.0)

[a] Sugar-free beverages; [b] Sugar-sweetened beverages

Supplemental Table 3: Model-based mean (95% confidence interval) anthropometric measures of Iowa Fluoride subjects by age 13- 17-year beverage cluster (n=365).

Variable	Age (years)	Cluster Group									
		100% Juice		Milk		Water/SFB		Neutral		SSB	
		F	M	F	M	F	M	F	M	F	M
n		27	14	16	35	37	33	71	30	42	60
Weight (kg)	5	19.5 (17.3, 21.8)	19.8 (16.6, 23.0)	21.0 (18.0, 24.0)	21.2 (19.2, 23.3)	21.5 (19.4, 23.5)	21.1 (19.0, 23.3)	20.3 (18.8, 21.7)	20.2 (17.9, 22.4)	21.2 (19.3,23.1)	21.7 (20.1, 23.2)
	9	29.5 (26.9, 32.2)	31.4 (27.5, 35.2)	33.0 (29.3, 36.7)	34.4 (31.8, 36.9)	36.2 (33.6, 38.7)	35.7 (32.9, 38.4)	32.6 (30.9, 34.3)	33.8 (30.9, 36.6)	33.9 (31.5,36.3)	35.8 (33.9, 37.8)
	13	49.7 (46.1, 53.3)	52.2 (47.0, 57.3)	56.3 (51.1, 61.5)	57.4 (53.7, 61.0)	61.1 (57.4, 64.7)	62.3 (58.3, 66.4)	53.6 (51.2, 56.0)	57.3 (53.3, 61.3)	59.5 (56.1,62.8)	58.8 (56.0, 61.5)
	17	61.0 (56.7, 65.2)	69.8 (63.2, 76.3)	69.6 (63.3, 75.8)	79.5 (74.6, 84.3)	71.4 (67.3, 75.6)	83.1 (77.9, 88.3)	62.4 (59.7, 65.1)	78.3 (73.0, 83.5)	70.3 (66.5,74.2)	79.3 (75.7, 82.9)
Height (cm)	5	110.8 (108.4, 113.2)	110.9 (106.9, 114.9)	111.9 (108.8, 114.9)	113.2 (110.7, 115.8)	111.5 (109.4, 113.5)	111.4 (108.8, 114.1)	109.6 (108.1, 111.1)	110.5 (107.7, 113.4)	111.4 (109.5, 113.3)	111.7 (109.8, 113.6)
	9	133.9 (131.5, 136.3)	136.3 (132.3, 140.3)	137.2 (134.0, 140.4)	139.3 (136.7, 141.9)	137.7 (135.7, 139.8)	138.7 (136.0, 141.3)	134.7 (133.2, 136.2)	135.9 (133.1, 138.8)	136.1 (134.1, 138.0)	138.4 (136.5, 140.3)
	13	161.8 (159.4, 164.2)	162.5 (158.6, 166.4)	163.6 (160.5, 166.7)	167.7 (165.1, 170.2)	162.6 (160.6, 164.6)	165.8 (163.2, 168.4)	159.8 (158.3, 161.3)	163.3 (160.5, 166.0)	161.8 (159.9, 163.7)	164.8 (162.9, 166.7)

	17	167.5 (165.1, 169.9)	177.9 (174.0, 181.7)	168.4 (165.3, 171.5)	181.8 (179.3, 184.4)	165.8 (163.8, 167.9)	180.3 (177.7, 182.9)	164.0 (162.6, 165.5)	177.0 (174.2, 179.8)	166.8 (164.9, 168.7)	179.5 (177.6, 181.4)
Body Mass Index	5	15.5 (14.1, 16.8)	15.9 (14.2, 17.6)	16.5 (14.7, 18.3)	16.2 (15.1, 17.3)	17.1 (15.9, 18.3)	16.5 (15.4, 17.6)	16.5 (15.6, 17.3)	16.0 (14.8, 17.2)	16.6 (15.5,17.7)	16.9 (16.1,17.7)
	9	16.5 (15.1, 17.8)	16.8 (15.1, 18.5)	17.9 (16.0, 19.7)	18.1 (17.0, 19.3)	19.2 (18.0, 20.4)	18.7 (17.5, 19.8)	18.1 (17.2, 18.9)	18.2 (16.9, 19.4)	18.6 (17.4,19.7)	18.7 (17.9,19.6)
	13	19.0 (17.6, 20.5)	19.8 (18.0, 21.5)	21.1 (19.2, 23.0)	20.4 (19.3, 21.6)	23.1 (21.7, 24.4)	22.5 (21.3, 23.8)	21.0 (20.1, 21.9)	21.4 (20.1, 22.7)	22.6 (21.4,23.8)	21.6 (20.7,22.5)
	17	21.8 (20.3, 23.3)	22.0 (20.2, 23.8)	24.3 (22.3, 26.3)	24.0 (22.7, 25.2)	25.8 (24.4, 27.2)	25.5 (24.1, 26.8)	23.1 (22.2, 24.1)	24.9 (23.5, 26.3)	25.1 (23.9,26.4)	24.6 (23.7,25.6)

[a] Sugar-free beverages; [b] Sugar-sweetened beverages
